# Lyophilized Avocado Paste Improves Corn Chips’ Nutritional Properties and Sensory Acceptability

**DOI:** 10.3390/foods13081220

**Published:** 2024-04-17

**Authors:** B. Shain Zuñiga-Martínez, J. Abraham Domínguez-Avila, R. Maribel Robles-Sánchez, J. Fernando Ayala-Zavala, Manuel Viuda-Martos, José Alberto López-Díaz, Mónica A. Villegas-Ochoa, Gerardo Torres-García, Gustavo A. González-Aguilar

**Affiliations:** 1 Centro de Investigación en Alimentación y Desarrollo A. C. Carretera Gustavo Enrique Astiazarán Rosas No. 46, Col. La Victoria, Hermosillo 83304, Sonora, Mexico; shain.zuniga21@gmail.com (B.S.Z.-M.); jayala@ciad.mx (J.F.A.-Z.);; 2 CONAHCYT—Centro de Investigación en Alimentación y Desarrollo A. C. Carretera Gustavo Enrique Astiazarán Rosas No. 46, Col. La Victoria, Hermosillo 83304, Sonora, Mexico; 3 Departamento de Investigación y Posgrado en Alimentos, Universidad de Sonora, Blvd. Luis Encinas y Rosales s/n, Col Centro, Hermosillo 83000, Sonora, Mexico; 4 IPOA Research Group, Agro-Food Technology Department, Instituto de Investigación e Innovación Agroalimentaria y Agroambiental (CIAGRO-UMH), Miguel Hernández University, 03312 Orihuela, Spain; mviuda@umh.es; 5 Departamento de Ciencias Químico-Biológicas, Instituto de Ciencias Biomédicas, Universidad Autónoma de Ciudad Juárez, Anillo Envolvente del PRONAF s/n, Ciudad Juárez 32310, Chihuahua, Mexico; joslopez@uacj.mx; 6 Centro de Investigación en Alimentación y Desarrollo, Circuito Gobernador C, Ney González # 10, Ciudad del Conocimiento, Tepic 63173, Nayarit, Mexico

**Keywords:** bioactive compounds, agroindustrial waste, functional properties

## Abstract

Avocado paste (AP) is an industrial byproduct and a potential source of bioactive compounds, so there is great interest in its valorization. The objective of the present study was to evaluate the effects of adding AP to corn chips regarding their nutritional profile and sensory acceptability. Three AP-supplemented corn chip samples were prepared (C-2%, C-6%, and C-10%), along with a control chip (C), whose total phenolics, flavonoids, antioxidant capacity, proximate composition, minerals, fatty acids, and sensory acceptability were evaluated. Regarding the content of phenolic compounds and flavonoids, significant increases were found between all samples (*p* < 0.05), particularly between C and C-10% (from 0.93 to 3.56 mg GAE/g dw and 1.17 to 6.61 mg QE/g dw, respectively). Their antioxidant capacity also increased significantly (*p* < 0.05) with all methods used (FRAP, DPPH, ORAC, and TEAC). Regarding the sensory analysis, no significant differences were found (*p* > 0.05) between C and C-2% in the parameters of smell, color, flavor, and overall acceptability; however, the texture of C-2% was better evaluated. The C-2% sample also had the highest acceptability; 82% of the participants mentioned that they would buy the C-2%, higher than the rest of the samples. These results suggest the feasibility of adding 2% AP as a strategy to improve the nutritional properties of corn chips without compromising their sensory acceptability; therefore, AP may be used as a food ingredient.

## 1. Introduction

The consumption of avocado has increased due to its high nutritional value and beneficial health effects [1]. Mexico is the main producer, with a third of world production in 2018. Mexican avocado is consumed fresh, used as a source of oil, or processed into “guacamole” purée, slices, packaged pieces, and dehydrated, while uses in the pharmaceutical and cosmetic industries are also common [2]. One of the biggest problems faced by avocado processing industries is the generation of large amounts of byproducts (seed, defatted pulp, and peel), as well as the negative effects of their inadequate handling, the cost of their transport and disposal, and the land required to store them [3], in addition to their causing greenhouse gas emissions [4]. Avocado-oil-producing industries, through the method of mechanical extraction of oil by cold pressing, produce a significant amount of byproducts, i.e., approximately 420 kg (defatted pulp, seed, and peel) per ton of fresh avocado processed [5]. These byproducts are wasted and lack commercial applications; however, they are a renewable, abundant, and economical source of bioactive compounds, such as fiber, carotenoids, tocopherols, phenolic acids (*p*-coumaric, ferulic, and protocatechuic acid), protein, and fatty acids, among others [6]. They could, therefore, be used for the formulation of new functional foods, thereby minimizing disposal costs and environmental impact and, at the same time, meeting the growing consumer demand for foods with beneficial health effects. This also promotes the transition towards a circular economy with the implementation of the zero waste policies proposed in the 2030 agenda of the United Nations [7].

The nutrients and bioactive compounds present in avocado and its byproducts have shown various biological activities, such as antioxidant and anti-inflammatory properties, which are relevant to the consumer’s health [8]. For example, Nguyen et al. [9] reported that the administration of *p*-coumaric acid (200 mg/kg of body weight) for 8 weeks to mice fed a high-fat diet improved their glucose homeostasis, an effect that was attributed to this compound’s ability to promote peripheral activation of AMPK, which responds to leptin-melanocortin signaling and regulates food intake. Likewise, Qi et al. [10] observed that the administration of ferulic acid (100 mg/kg) for 8 weeks to rats with diabetic nephropathy improved renal tissues and pathological lesions and increased the activities of antioxidant enzymes (SOD, CAT, and GPx). Other authors evaluated the impact of protocatechuic acid on the sequelae of metabolic syndrome in rats fed a high-fat diet for 14 weeks; the administration of this compound decreased body weight, insulin resistance, oxidative stress, and inflammation [11]. Because of its content of bioactive compounds, avocado byproducts have also been evaluated for various uses and applications, such as a lipid-peroxidation-inhibiting food preservative [5]. These uses could minimize their economic and environmental impacts, while supporting the search for additional functional ingredients that can benefit the consumer’s health [12]. We have previously determined the feasibility of supplementing baked goods with promising results, such as with corn chips supplemented with mango peel, extruded papaya peel, and mango; muffins supplemented with apple, grape, carrot, and orange pomace; and cookies supplemented with grape pomace, pomegranate peel, and prickly pear peel [13]. This suggests the viability of supplementing baked foods with fruit byproducts of different sources and at varying percentages. Baked foods are optimal candidates to be supplemented with vegetable byproducts since they can improve their nutritional, bioactive, and sensory properties, and are widely consumed worldwide. For example, corn chips account for 80% of corn-based snacks, but they are generally made from processed flour, resulting in a high glycemic index product and, if fried, they can also be rich in fats/oils [14]. Because of this, supplementing them with vegetable byproducts can increase their fiber and phenolic compound content and antioxidant capacity while also preserving or increasing their sensory acceptability [15], although studies that focus on the effects of avocado byproducts are lacking. The present study aimed to develop baked corn chips supplemented with avocado paste (AP) and evaluate their physicochemical properties, proximate composition, bioactive compound profile, and sensory acceptability. We hypothesize that adding AP to baked corn chips will positively affect the physicochemical properties, proximate composition, bioactive compound profile, and sensory acceptability of the resulting products.

## 2. Materials and Methods

### 2.1. Materials

The chemical reagents and solvents used in the present work are shown in Table 1. AP used was generated in an avocado processing plant in Uruapan, Michoacan, Mexico. It consisted of defatted pulp, peel, and seed (in unspecified proportions), which were left over after oil extraction. Industrially generated AP has been previously characterized and shown to contain fatty acids, phenolic acids (ferulic, protocatechuic, and *p*-coumaric acids), carotenoids, and tocopherols [6]. AP was shipped to the laboratory, where it was immediately frozen at −30 °C, freeze-dried (FreeZone 6 Liter Benchtop, Labconco Corporation, Kansas, MO, USA) for 72 h, and sieved (<250 μm mesh) until a fine yellow-brown flour was obtained. The flour was stored in plastic bags until use.

### 2.2. Preparation of Corn Chips Supplemented with AP

All formulations were prepared on the same day and contained increasing percentages of AP flour (2, 6, and 10%) that were used to substitute corn flour. A control sample was also prepared, containing only commercial corn flour (Minsa, Hermosillo, Mexico) (C). The percentages of AP added were established based on preliminary tests, which showed that more than 10% AP yielded products with unacceptable sensory properties. Ingredients were placed in a kneader (Oster pedestal mixer, Oster, Mexico City, Mexico) and mixed at low speed for 3 min. Doughs were manually pressed, extended (2 mm thickness), and cut into triangular shapes. Pieces were preheated for 1 min on both sides at 200 °C in an electric pan and then baked in an electric oven (National MFG. Co., Lincoln, NE, USA) for 7 min at 180 °C. Corn chips were then cooled and stored in plastic bags at 25 °C.

### 2.3. Physicochemical and Proximate Analyses

Sample color was analyzed by means of a Minolta digital colorimeter (Model CR-400, Konica Minolta Sensing Inc., Osaka, Japan) according to the L*, a*, and b* color system; a* and b* data were then used to calculate chroma and hue angle. Corn chip hardness was measured with a texturometer (Model TA-XT2, Stable Micro System, Godalming, Surrey, UK) and expressed as the maximum force (N) needed to break the chip [15]. Water activity (A_w_) was determined using a portable PawKit water activity meter (Aqualab, Pullman, WA, USA) at 25 °C (25 °C) using a sample of ground corn chips [16]. Color and texture were measured ten times, while A_w_ was measured in triplicate.

A proximate analysis of the corn chips was carried out, where moisture (AOAC 950.46), ash (AOAC 923.03), total protein (AOAC 984.13), total fat (AOAC 960.39), and total fiber (AOAC 991.43) were quantified according to the standard analytical methods described by the procedures of the Association of Official Analytical Chemists International (AOAC International) [17]. Total carbohydrates were calculated by difference [18].

### 2.4. Methanolic Extraction

A methanolic extract of the corn chips (control and supplemented with AP) was prepared to determine their phenolic content and antioxidant capacity according to the methodology described by Palafox-Carlos et al. [19]. For this, 1 g of ground sample was homogenized with 20 mL of methanol–water (80:20, *v*/*v*), sonicated for 30 min in a sonicator (Bransonic Ultrasonic Co., Danbury, CT, USA), and centrifuged (Beckman Coulter, Palo Alto, CA, USA) at 10,000 rpm for 15 min at 4 °C. The supernatant was recovered, and the precipitate was re-extracted twice more with 10 mL of the same solvent and processed under the same conditions. The supernatants obtained were combined, filtered through Whatman No. 1 paper, and stored at −20 °C until later use.

### 2.5. Quantification of Phenolic Compounds and Flavonoids

Total phenolic compounds were quantified using the Folin–Ciocalteu method [20]; a standard curve of gallic acid was used to calculate these values, according to their absorbance at 765 nm. Data were expressed as mg gallic acid equivalents (GAE)/g dry weight (dw). Flavonoid content was determined using a standard quercetin curve; absorbance was measured at 496 nm and was expressed as mg of equivalents of quercetin (QE)/g dw [21]. Both analyses were performed in triplicate; absorbances were read in a FLUOstar Omega spectrophotometer (BMG Labtech, Durham, NC, USA).

### 2.6. Antioxidant Capacity

Antioxidant capacity was determined using four different methods; TEAC (2,2′-azinobis-3-ethyl-benzo-thiazoline-6-sulfonic acid) using the methodology of Re et al. [22]; ORAC (oxygen radical absorption capacity) according to the methodology of Robles-Sánchez et al. [23]; DPPH radical scavenging activity according to Brand-Williams et al. [24]; and the FRAP assay according to the methodology of Benzie and Strain [25]. Results of all these methods were expressed as mg of Trolox equivalents (TE)/g dw. Antioxidant capacity analyses were performed in triplicate; absorbances were read in a FLUOstar Omega spectrophotometer (BMG Labtech).

### 2.7. Fatty Acid Profile

To identify the fatty acids present in the samples, a total lipid extraction was carried out following the methodology of Folch et al. [26]. The lipid phase was obtained, and the fatty acids were methylated as previously described [17]. After obtaining the methylated fatty acids, they were analyzed in an HP 6890 gas chromatograph with a flame ionizing detector following the methodology of Botella-Martínez et al. [27]. The analyses were performed in triplicate and expressed as g of fatty acid/100 g of oil. From these, the atherogenic index (*AI*) (Equation (1)) and thrombogenic index (*TI*) (Equation (2)) were obtained as described by Ulbricht and Southgate [28]:

(1) $$AI=\frac{C12:0+\left(4\times C14:0\right)+C16:0}{\Sigma MUFA+\Sigma n6+\Sigma n3\;},$$

(2) $$TI=\frac{C14:0+C16:0+C18:0}{\left(0.5\times \Sigma MUFA\right)+\left(0.5\times \Sigma n3\right)+\left(\frac{\Sigma n3}{\Sigma n6}\right)},$$

where *AI*: atherogenic index; *TI*: thrombogenic index; ΣMUFA: total concentration of monounsaturated fatty acids; Σ*n*6: total concentration of omega−6 fatty acids; Σ*n*3: total concentration of omega−3 fatty acids; C12:0: concentration of lauric acid; C14:0: concentration of myristic acid; C16:0: concentration of palmitic acid; C18:0: concentration of stearic acid.

### 2.8. Mineral Content

The mineral content of corn chips was determined using inductively coupled plasma mass spectrometry (ICP-MS) in a Shimadzu MS-2023 apparatus, following the methodology described by Muñoz-Bas et al. [29]. The analyses were performed in triplicate, and the mineral content was expressed as mg/100 g.

### 2.9. Sensory Analysis

Approval was obtained from the Institutional Ethics Committee of the Research Center for Food and Development (CEI/032-1/2021). Panelists were recruited and briefed about the nature of the study and the composition of the samples; relevant food allergies were also specifically consulted beforehand. They were also informed that their participation was voluntary and that they were free to leave at any time. Oral consent was then obtained in the presence of at least two research team members, who served as witnesses. This procedure aligns with our Institutional Ethics Committee’s guidelines, based on the National Guide for the Integration and Operation of Research Ethics Committees, the General Health Law in Health Research, and the ethical principles outlined in the Declaration of Helsinki.

A sensory analysis of the AP-supplemented samples was carried out, following the methodology of Vollmer et al. [30]. Evaluations were made at room temperature and under white fluorescent light. Panelists were instructed to rinse their mouths with water and consume a piece of unsalted soda crackers between samples to minimize any residual taste. A 10 cm seven-point hedonic scale was used, which ranged from “I dislike it a lot” to “I like it a lot”, with a neutral rating of “I neither like it or I dislike it” in the center (4). These ratings determined each sample’s acceptability according to five parameters (smell, color, taste, texture, and overall acceptability). Panelists were also asked to express their purchase intention (yes or no), and a blank space was left for comments. Samples were labeled with random three-digit codes, and provided in a random order on white plates, divided into four equal parts.

### 2.10. Experimental Design and Statistical Analysis

The experimental factors were concentrations (0, 2, 6, and 10%), using a completely randomized design. The normality of the data was tested using the Kolmogorov–Smirnov test. A matrix correlation was used to determine correlations between phenolics, flavonoids, and antioxidant capacity. All measurements were carried out as standalone experiments and performed in triplicate. Data were expressed as mean ± standard error (SE). One-way ANOVA was conducted to assess significant differences in the data at *p* < 0.05. For the sensory analysis, a completely randomized design with repeated measures was used with a one-way ANOVA statistical analysis; the factors were the percentages of AP added to the corn chips; the variables were the values of smell, color, flavor, texture, and overall acceptability, and determined using the Tukey–Kramer test for comparison of means, with a significance level *p* < 0.05, using the NCSS 2012 software (NCSS, LLC, Kaysville, UT, USA).

## 3. Results

### 3.1. Physicochemical and Proximate Analyses

**Figure 1:**
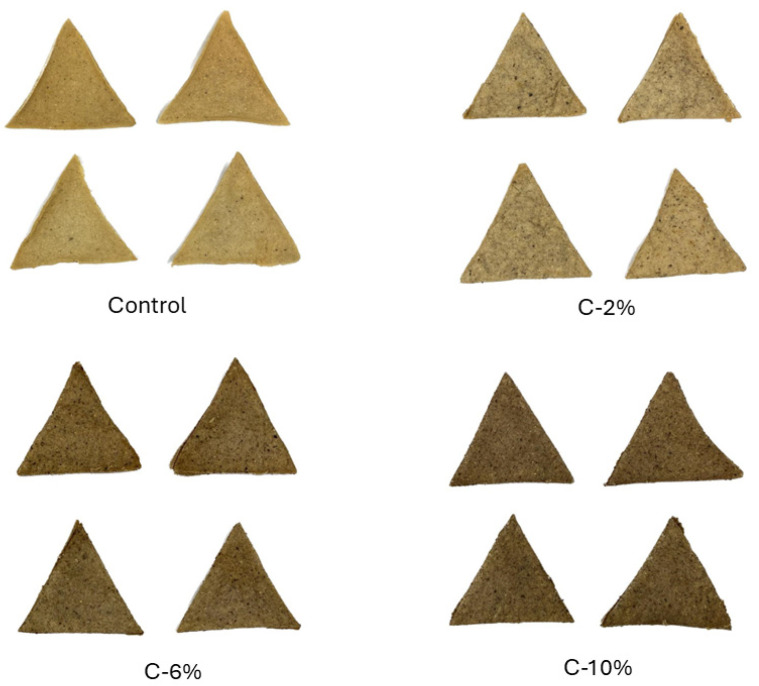
Appearance of corn chips at AP substitution levels of 0, 2, 6, and 10%.

Figure 1 shows the appearance of corn chips, while Table 2 shows the results of all samples’ physicochemical and proximate composition analyses. Color measurements showed significant differences among all samples (*p* < 0.05); specifically, L* and Hue° values are inversely proportional to the percentage of AP added, indicating that the samples become slightly darker as the percentage of AP increases. Chromaticity values of the samples ranged between 23 and 32, indicating that the chips had an opaque red-yellow saturation, while the Hue° of the samples ranged between 74 and 87, positioning them in the upper quadrant of the color wheel. The hardness of all samples showed significant differences (*p* < 0.05) between C and AP-supplemented samples, thereby showing that supplementing them with AP modifies their texture. The sample with the highest percentage of AP (10%) had the highest hardness, suggesting that the force needed to break the chips increased. Regarding A_w_, significant differences can be observed between C-2% and C-10%, with no significant differences between C and C-6%. Moisture varied but remained under 5% for AP-supplemented samples, in contrast to C, which had a higher value. Ash content varied by approximately 0.3% between all samples. Protein content was similar among most samples, except for C-2%, which had a slightly lower value. Regarding lipids, C-10% had the highest content, followed by C and C-6% and C-2%. Interestingly, fiber content increased with the amount of AP added, although total carbohydrates decreased; carbohydrates were the main component in all samples, with values ranging from 77 to 84%.

### 3.2. Minerals

The mineral content of corn chips is shown in Table 3. Although minerals were present in the main ingredient (corn flour), it can be observed that supplementing it with AP increased their concentrations. Most notably, C-10% presented significantly higher values (*p* < 0.05) of most minerals, as compared to the other samples, including Ca, Cu, K, Na, and P. There were significant differences in Mn content between C-6% and C-10%, as compared to C and C-2%. In terms of Zn content, C had a higher content than the other samples.

### 3.3. Fatty Acid Profile

A total of 17 fatty acids were identified in C and AP-supplemented samples, as shown in Table 4. The most abundant fatty acids in the samples were, in decreasing order, linoleic acid (C18:2 n-6), oleic acid (C18:1 n-9), and palmitic acid (C16:0). The C-10% samples showed significant differences (*p* < 0.05), as compared to the other samples regarding palmitic and oleic acids, while C showed significant differences (*p* < 0.05) in terms of linoleic acid (C18:2 n-6), as compared to AP-supplemented samples. C also had a higher (*p* < 0.05) content of omega-6 faffy acids as compared to the other samples; there were no significant differences in terms of omega-3 content. Regarding the content of monounsaturated (MUFA) and saturated (SFA) fatty acids, C-10% had higher values than the other samples. Atherogenic and thrombogenic indices also increased as the levels of AP substitution increased (*p* < 0.05).

### 3.4. Phenolic Compounds, Total Flavonoids, and Antioxidant Capacity

Bioactive compounds in AP-supplemented samples were analyzed, and the results are shown in Table 5. Total phenolic compounds varied significantly (*p* < 0.05); most notably, C-10% samples had the highest content, approximately threefold higher than C. A trend was also observed, where their concentration increases linearly with the amount of AP. The antioxidant capacity of AP-supplemented samples was evaluated using four techniques: TEAC, FRAP, DPPH, and ORAC. There were significant differences between C and all AP-supplemented samples, but the magnitude of the change varied by method; for example, TEAC and DPPH values approximately doubled, FRAP increased by 50%, and ORAC tripled when comparing C with C-10%. Table 6 shows a correlation matrix between phenolic compounds, flavonoids, and antioxidant capacity (TEAC, FRAP, DPPH, and ORAC). It can be observed that these variables correlate positively and significantly (*p* < 0.05) with each other, but the best fit was between phenolic compounds with FRAP and ORAC, as well as ORAC and FRAP.

### 3.5. Sensory Analysis

**Figure 2:**
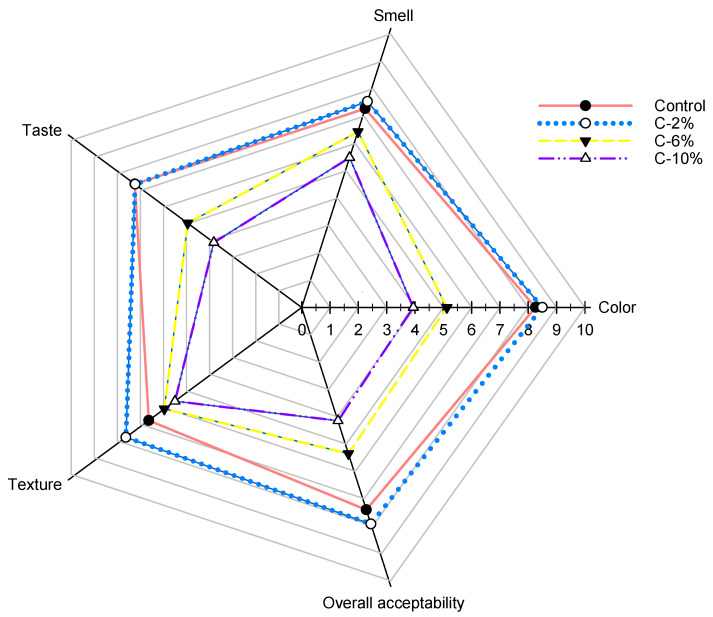
Radar graph of sensory analysis of AP-supplemented corn chips.

The sensory analysis results of AP-supplemented samples are shown in Figure 2, wherein 109 untrained panelists participated (66 women and 43 men, aged between 18 and 58 years), all of which were students and staff of CIAD and the University of Sonora (Hermosillo, Mexico). It can be seen that there are no significant differences in terms of taste, smell, color, and overall acceptability between C and C-2%, although the panelists indicated that the C-2% sample had a better texture. This improvement may be due to interactions between the components of corn flour and AP (water, starch, and protein complexes) [31]. C-6% and C-10% also showed significant differences; as the percentage of AP increased, the sample rating tended to decrease on all parameters evaluated.

**Figure 3:**
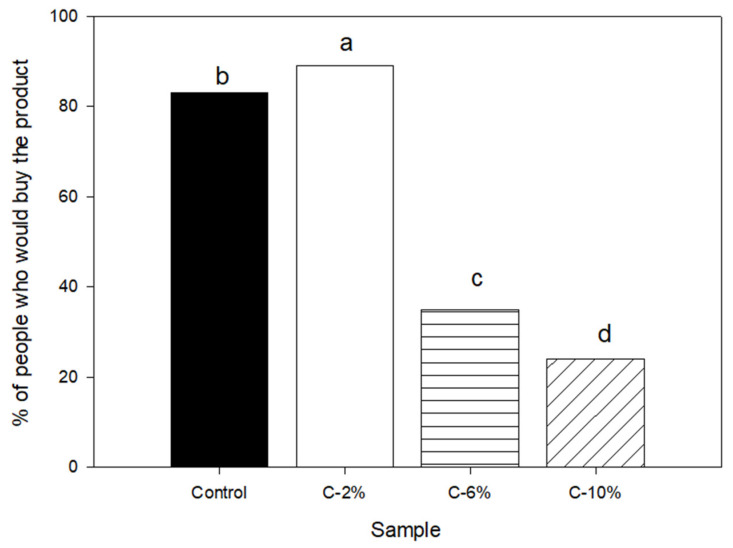
Purchase intention of corn chip samples. Different letters represent significant differences at *p* < 0.05.

The panelists were also asked if they would buy the samples evaluated; results are shown in Figure 3. There are significant differences between all samples; 82% of panelists indicated that they would buy the C-2% samples, while only 76% would buy C, 32% would buy C-6%, and only 22% would buy C-10%. These results could be because the intrinsic properties of a product, such as its sensory properties, are determining factors for the choice and acceptance of a product.

**Figure 4:**
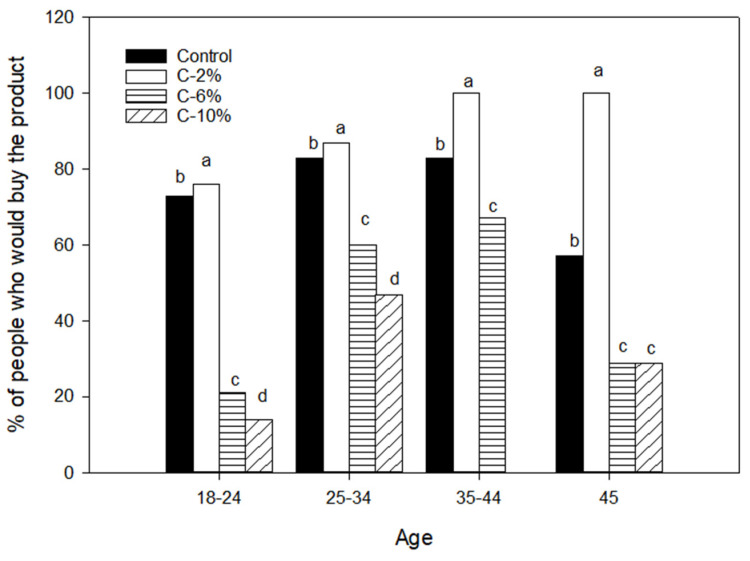
Purchase intention of corn chips samples by age. Different letters on each age group represent significant differences at *p* < 0.05.

The percentage of panelists who would buy the product was further analyzed by age; results are shown in Figure 4. C-2% samples had the highest preference in all age groups, followed by C, C-6%, and C-10%. According to these results, favorable changes are observed when supplementing with AP; given the main objective of this work, the performance in the acceptability criteria assumes the greatest importance, which allows us to propose that corn chips supplemented with 2% AP was in fact the best developed formulation.

**Figure 5:**
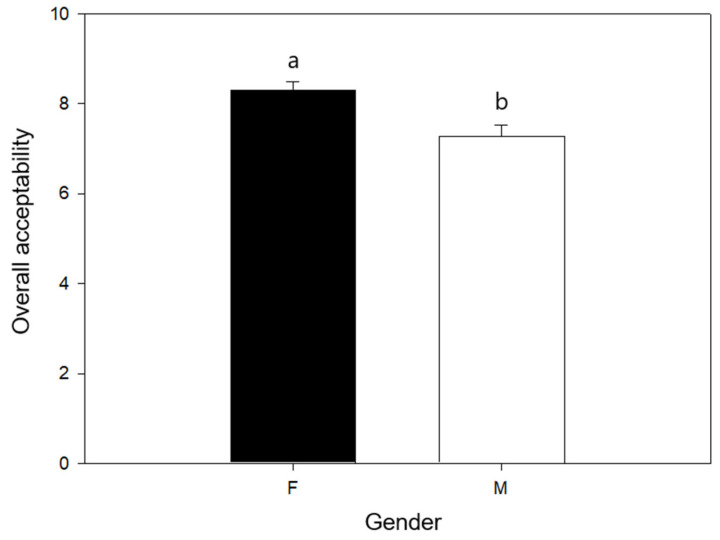
Overall acceptability by gender (F: female, M: male) of C-2%. Different letters represent significant differences at *p* < 0.05.

The acceptability of C-2% samples was finally evaluated by gender, as shown in Figure 5. It is apparent that women expressed a significantly higher score for these samples, although men still showed a positive evaluation.

## 4. Discussion

We have previously reported the physicochemical properties of industrially generated AP [6]. Some are particularly interesting and can be attributed to its content of peel (such as its dark color and increased fiber content) or of seed (such as its fatty acid profile). Moreover, its organoleptic properties are also likely to have an influence on consumer perception, once incorporated into a given food, since the color, texture, and taste of the final product may change due to the macronutrient and phenolic composition of AP.

Regarding the physicochemical properties, specifically the change in color of the supplemented corn chips, this can be attributed to the brownish color of AP, which itself may be due to the content of chlorophylls, carotenoids, and tannins present in avocado peel [32]. As for chromaticity, similar findings have been documented in other studies; for example, Zepeda-Ruiz, Domínguez-Avila, Ayala-Zavala, Robles-Sánchez, Salazar-López, López-Díaz and González-Aguilar [15] supplemented corn chips with mango peel, while Mehta et al. [33] supplemented bread and muffins with tomato byproducts. These authors also report a decrease in luminosity as the percentage of byproduct added increased, leading to progressively darker samples. Consumers prefer chips with tonalities that suggest at least some level of toasting; this makes them more visually attractive and can be achieved in the food industry using artificial colors to enhance this characteristic [34]. Our results suggest that supplementing corn chips with AP imparts them with a similar toasted appearance; thus, changing the percentage added could be used as a strategy to modulate their desired appearance without the need for artificial ingredients. We observed that the addition of AP to corn chips had a significant impact on the physicochemical properties of the final products, most notably in terms of color, macro- and micronutrient profiles, and texture.

Regarding the changes in texture, a similar effect has been reported in corn chips supplemented with 5 and 10% mango and Hibiscus flower husk [35]. Texture is important in determining the consumer’s preference for crunchy foods [36]. Likewise, a food’s macrostructural properties (texture) impact the consumer’s energy intake and metabolic processes in response to ingested nutrients since different textures can trigger hormonal responses that influence later parts of the satiety cascade [37]. Therefore, an adequate food texture can be significant for more than just its organoleptic properties. As previously mentioned, it can also induce health effects on the consumer, such as appetite control and food intake [38].

The addition of AP on physicochemical parameters like the ones analyzed here can be objectively documented, such as with the Lab color space, but it can also affect its subjective appreciation by the consumers, such as modifying their preference due to the chips’ color. Other physicochemical parameters can also change due to interactions between the main ingredient (corn flour in this case) and the added AP, such as texture or changes in taste. Thus, it is apparent that supplementing corn chips with AP must take into consideration such physicochemical changes and how they could be perceived by the consumer.

A_w_ values found herein are lower than those reported by Mayo-Mayo, Navarrete-García, Maldonado-Astudillo, Jiménez-Hernández, Santiago-Ramos, Arámbula-Villa, Álvarez-Fitz, Ramirez, and Salazar [35] in corn chips supplemented with mango and Hibiscus flower husk, which may be attributed to differences in the food matrix of the byproduct and type or drying method used, since they were dried in a convection oven (40 °C, 24 h), while those used here were freeze-dried, which is a more effective method for water removal. A_w_ is a variable that can enable or hinder microbial growth in food; lower values are therefore useful to increase a food’s resistance to pathogen proliferation. It has been reported that A_w_ should be around 0.60, sufficient to inhibit the growth of microorganisms [39].

Minerals have specific functions in the physiological and biochemical processes of the human body. Nine minerals were identified and quantified in the samples analyzed in this study that are necessary for various metabolic processes; dietary Fe is vital for cell growth and proliferation, Cu for the synthesis of hemoglobin [40], while others like Mg and Ca are necessary to regulate enzyme activities and various homeostatic functions (including oxidative stress, inflammation, specific immunity, acid-base balance and others) [41]. P is an essential component of bone mineral, cell membranes, and nucleic acids, while K plays a fundamental role in fluid homeostasis, muscle contraction, nerve impulses, and blood pressure reduction [42]. Na is a key electrolyte for regulating blood pressure, blood volume, and blood osmolality; however, a high intake is related to cardiovascular disease and an increased risk of hypertension [43]. The present samples contained low Na and high K, suggesting a favorable profile, particularly when comparing them with traditional fried and salted chips, whose Na content is regularly high and low in K.

Commercial corn chips have a high content of SFA because they are most commonly fried; for this reason, studies have been carried out to improve the ratio of saturated-to-monounsaturated fatty acids and the specific profile of these molecules. A favorable decrease in omega-6 is observed, mainly due to a decrease in linoleic acid, while the presence of omega-3 can be attributed to α-linolenic acid, since this was the main omega-3 present in all samples. Although there were no significant differences in the sum of omega-3, the aforementioned α-linolenic acid did show a significantly increased concentration. The optimal n6/n3 ratio should range from 1:1 to 5:1; however, in Western diets, which are characterized by high n6 intake, the range is modified from 10:1 to 20:1, thereby promoting a pro-inflammatory state [44,45]. The n6/n3 ratio takes into account the detailed composition of PUFAs in a food product and how it can impact the consumer’s health; its importance has been known and considered for more than 30 years [46] because, in the Western diet, this range has increased considerably. Modifying it can positively affect cardiovascular health, e.g., reducing oxidative stress, among others. The samples analyzed herein had a n6/n3 ratio of approximately 42 in C; when AP was added, this ratio decreased significantly, which is attributed to a decrease in linoleic acid (n6) and an increase in linolenic acid (n3). Regarding the atherogenic and thrombogenic indices, these were within the range of comparable foods of vegetable origin like cereals; foods low in these indices have beneficial effects on human health because they help to delay or prevent the risk of cardiovascular diseases; thus, decreasing them can be considered ideal [47,48]. It would be important to evaluate the atherogenic and thrombogenic indices in other products with partial substitution of vegetable byproducts to establish a comparison with products on the market, and thus conclusively demonstrate that they can have a positive health impact. The lipid content of AP has been reported to contain approximately 58% of MUFAs, with oleic acid being the most abundant, followed by linoleic acid [6], all of which are present in avocado pulp and have been previously studied. For example, Dreher and Davenport [49] reported that avocado consumption in a healthy population positively affected blood lipids regulation, such as decreased total cholesterol, LDL, and triacylglycerols, while HDL cholesterol increased. These results suggest that avocado byproducts (such as AP), which contain said MUFAs with positive health effects, may be used as food additives that increase a product’s nutritional content.

As for the protein and fiber content, AP-supplemented samples had a higher protein content than C; these macronutrients exert various effects on the consumer, such as delaying carbohydrate digestion, resulting in a lower glycemic index, as well as an additive effect on satiety by reducing energy intake and energy expenditure, increasing chewing time, increasing bolus viscosity, and modulating the secretion of satiety-related hormones (such as GLP-1 and PYY) [50]. Others have reported that adding vegetable byproducts to snacks increases their fiber and protein content [51], similar to the results reported herein. It should also be noted that total carbohydrate content decreased slightly due to the partial replacement of corn flour with AP; therefore, the profile of these macronutrients could further contribute to slowing down their digestion and absorption, thus modifying the consumer’s postprandial glycemic responses [52]. Based on the samples’ proximate composition, it is possible to suggest that AP may be considered a functional food ingredient with potential applications in bakery products to improve their nutritional quality. The fatty acids present in AP also present the option not only to produce low-fat chips but also to enrich corn chips that could exert benefits to human health; however, conclusively demonstrating this requires additional experimentation.

Our results show a higher phenolic content than that reported in corn chips supplemented with Hibiscus flower husk [35]. Another study reports that phenolic compounds increased in cookies supplemented with prickly pear shells in parallel with the added byproduct [53]. Said cookies had similar values to our samples; however, the prickly pear shells were obtained under controlled laboratory conditions, while the AP used herein is derived from the avocado processing industry. The content of total flavonoids also showed significant differences (*p* < 0.05) between all samples; in particular, C-10% had the highest content, which was fivefold higher than that of C. It has been reported that similar compounds can decrease free radicals and the risk of some non-communicable diseases, due in part to their hypoglycemic and hypolipidemic effects [54]. These compounds could exert important health effects on the consumer.

Since an increase in phenolic compounds can be observed on all supplemented samples, even at the lowest percentage, this indicates that AP retains significant amounts of these and possibly other bioactive compounds that confer its antioxidant capacity, and which were not extracted in their entirety during processing, despite the mechanical stress to which avocados are subjected during this stage. A similar behavior was found in cookies supplemented with prickly pear shells, where an increase was documented in response to adding a higher percentage of this byproduct [53]. The observed increase in antioxidant capacity may be due to the specific bioactive compounds present in AP-supplemented samples, which appear to have a better affinity with these antioxidant capacity methods. The content of total phenolic compounds and flavonoids was significantly correlated with antioxidant capacity as determined by four different methods (ABTS, DPPH, ORAC, and FRAP). These results indicate a relationship between the concentration of these compounds and their ability to scavenge free radicals. Phenolic compounds present in corn chips supplemented with AP may be significant contributors to their antioxidant capacity; phenolic acids like ferulic, protocatechuic, and *p*-coumaric acids, in addition to carotenoids and tocopherols, have been identified in AP [6]. Since these compounds are recognized for their significant roles as antioxidants, we propose that the increases in the chips’ antioxidant capacity can be attributed to them. The antioxidant capacity of bioactive compounds present in food or in the human body at very low concentrations can delay, control, or prevent oxidative processes that lead to the deterioration of food quality or the appearance and spread of some non-communicable diseases in the consumer [55]. The increase in antioxidant capacity found in our samples is thus an important indicator of their potential as health promoters, and which can be attributed to the presence of bioactive compounds from AP; other authors have reported something similar in different food matrices [56].

When performing a sensory analysis, panelists stated that C-10% samples had a harder texture and a bitter taste, similar to the results reported herein, which may be due to the higher fiber content of these samples [57,58]. They could also have negatively perceived the color of the samples with the highest AP percentage since their darker color could have suggested that they were burned or baked for too long. The ratings could also be reduced because the local commercially available products tend to be produced with bleached, refined flours, which have a lighter color [59]. Theagarajan, Malur Narayanaswamy, Dutta, Moses, and Chinnaswamy [59] report results similar to the present ones; they found that as the percentage of grape pomace added to cookies increased, it lowered their acceptability, where the cookie with 8% grape pomace obtained the lowest rating, as compared to the one with 6%. In the present study, C-2% samples had a higher acceptance than C-6% and C-10% and were also similar or better than C. Others report that when analyzing grape-pomace-supplemented muffins, the panelists mentioned a gritty sensation, as well as a sweet and intense flavor as the percentage added increased; the best-rated muffins were those that contained 15%, which stood out for their sweet taste, as compared to the ones that contained 25% and the control [60]. These results differ from those found herein, which may be because AP contains tissues with bitter flavors, such as peel and seed; thus, the highest ratings were obtained at lower percentages (C-2%), where the bitter compounds cannot be perceived as strongly, in addition to the previously mentioned improvement in texture, similar to the results reported herein. Likewise, Imeneo et al. [61] supplemented biscuits with up to 10% lemon peel, which acquired a bitter taste attributed to the peel’s fibrous components, although they still had adequate consumer acceptability. Therefore, the results of the present study indicate that the addition of AP at low percentages could produce corn chips with improved sensory properties.

Sensory cues like color, smell, taste, and texture are considered the most critical determinants of food perception and selection. The impact of gender difference, consumer hunger, and other personal factors, such as somatotype, preference, and frequency of purchase of baked products have been considered [62]. Sensory evaluation data were further analyzed, regarding the products’ overall acceptability by gender, where women showed a greater preference than by men. This could be associated with women being more health-conscious than men, since they tend to consume more healthy products and recognize certain characteristics. These gender differences have been previously reported in consumer studies, where they produced artisanal breads and cakes filled with jam [63] and found that men rated these products more sympathetically than women. Moreover, Hayes et al. [64] report that healthy women have a greater preference for saltier foods than men, highlighting the importance of considering male/female differences in taste preference in the sensory evaluation of novel foods.

Appearance attributes are key in influencing taste and the decision to eat as they shape the consumer’s first impression of a food product. Thus, increasing the percentage of AP beyond a certain threshold negatively influenced the chips’ appearance and the consumers’ purchase intention. However, other studies have also shown that consumer opinion can be positively influenced when they are made aware of the nutritional characteristics of a product [65].

Factors that affect consumer taste have been shown to differ by age, with younger consumers tending to value the hedonic scale and price more when shopping, while older consumers prioritize the health benefits of a product; likewise, young adults are more willing to accept new products [66]. The addition of AP in corn chips provided results where a significant increase in its physicochemical and sensory properties is observed.

The substitution of AP in corn chips at different percentages attenuated the nutritional attributes; however, at a higher percentage of supplementation, the sensory and hardness characteristics were compromised, which may be due to the peel and seed present in AP. From a technological point of view, one of the main aspects to consider in turning AP into a food ingredient is the proper drying method (such as freeze-drying as used here), since this can significantly influence the attributes of the dough and the resulting chips. In addition, the percentage of substitution is another important factor in achieving a good balance in fiber enrichment and technological quality, since quality parameters can be compromised. More research is needed to define the effect of AP on the health of the consumer; this could represent a valuable approach to developing dietary strategies for treating certain conditions like diabetes, obesity, and metabolic syndrome, among others. The functionality of these new formulations must be validated in vivo to continue generating knowledge in this area.

## 5. Conclusions

The present study reports the physicochemical properties, proximate composition, profile of bioactive compounds, and sensory acceptability of corn chips supplemented with varying percentages of avocado paste. The addition of avocado paste significantly changed the texture and water activity. An improved nutritional profile was also documented regarding the chips’ lipid profile and fiber content, which could potentially benefit the consumer. There was also an increase in bioactive compounds, which confer antioxidant capacity to the samples. The supplemented corn chips have a higher concentration of certain minerals (such as K) and fatty acids (such as oleic, palmitic, and linoleic), as well as a higher concentration of total monounsaturated fatty acids. Corn chip samples supplemented with 2% avocado paste had the greatest acceptance among panelists, particularly women over 35 years old. According to instrumental analyses and consumer acceptability, adding avocado paste at 2% maintains or increases the nutritional and organoleptic properties of corn chips. Further experimentation is required to validate their potential in vivo health effects and determine their possible biological potential on the consumer’s health.

## Figures and Tables

**Table 1:** Chemical reagents and solvents.

Reagent/Solvent	Supplier
Folin–Ciocalteu’s phenol reagent	Sigma-Aldrich (St. Louis, MO, USA)
2,2-diphenyl-1-picrylhydrazyl (DPPH)	
Phenolic standards (gallic acid and quercetin)	
Sodium carbonate > 99%	
Sodium chloride ≥ 99.5%	
TPTZ (2,4,6-tripyridyl-S-triazine)	
Trolox (6-hydroxy-2,5,7,8-tetramethyl-chroman-2-carboxylic acid)	
ABTS (2,2′-azino-bis-(3-ethylbenzothiazolne-6-sulfonic acid) diammonium salt)	
Ethanol (HPLC grade)	JT Baker (Phillipsburg, NJ, USA)
Hexane (HPLC grade)	
Acetone (HPLC grade)	

**Table 2:** Physicochemical and proximate analyses of corn chips supplemented with avocado paste (AP).

Variable	Control (C)	C-2%	C-6%	C-10%
L*	68.49 ± 8.74 ^d^	54.07 ± 5.34 ^c^	50.14 ± 6.37 ^b^	43.70 ± 7.58 ^a^
C*	23.55 ± 2.58 ^a^	29.43 ± 2.62 ^b^	32.01 ± 2.45 ^d^	26.27 ± 2.45 ^c^
Hue°	87.10 ± 2.23 ^d^	79.08 ± 1.91 ^c^	74.41 ± 2.48 ^a^	77.62 ± 1.68 ^b^
Hardness (N)	18.77 ± 0.02 ^c^	13.74 ± 0.25 ^a^	16.38 ± 0.13 ^b^	19.30 ± 1.52 ^d^
Water activity (A_w_)	0.11 ± 0.01 ^a^	0.18 ± 0.00 ^c^	0.10 ± 0.00 ^a^	0.16 ± 0.00 ^b^
Moisture (%)	8.17 ± 0.10 ^a^	3.65 ± 0.01 ^b^	1.72 ± 0.03 ^c^	4.33 ± 0.08 ^b^
Ash (%)	1.62 ± 0.01 ^c^	1.52 ± 0.01 ^b^	1.63 ± 0.01 ^c^	1.82 ± 0.01 ^a^
Protein (%)	7.76 ± 0.03 ^a^	7.67 ± 0.01 ^b^	7.71 ± 0.00 ^a^	7.75 ± 0.01 ^a^
Lipids (%)	2.98 ± 0.11 ^c^	2.08 ± 0.06 ^d^	2.64 ± 0.00 ^b^	3.62 ± 0.09 ^a^
Fiber (%)	2.72 ± 0.14 ^a^	3.38 ± 0.13 ^b^	4.38 ± 0.00 ^c^	5.29 ± 0.18 ^d^
Carbohydrates (%)	83.79 ± 0.19 ^a^	81.92 ± 0.12 ^b^	81.97 ± 0.19 ^a^	77.16 ± 0.10 ^c^

Values are expressed as % dw. Data are shown as the mean ± SD of three independent experimental runs. Different letters in each row represent significant differences at *p* < 0.05.

**Table 3:** Effect of partial substitution of avocado paste in corn chips on mineral content.

Variable	Control (C)	C-2%	C-6%	C-10%
Ca	235.41 ± 2.10 ^d^	243.51 ± 4.46 ^c^	260.87 ± 3.34 ^b^	284.90 ± 3.36 ^a^
Cu	0.07 ± 0.01 ^c^	0.20 ± 0.01 ^b^	0.20 ± 0.02 ^b^	0.26 ± 0.02 ^a^
Fe	10.49 ± 0.58 ^a^	9.13 ± 0.02 ^b^	9.17 ± 0.13 ^b^	9.48 ± 0.23 ^b^
K	216.56 ± 2.00 ^d^	249.58 ± 4.44 ^c^	261.69 ± 1.59 ^b^	312.60 ± 2.59 ^a^
Mg	101.48 ± 0.52 ^b^	104.14 ± 0.22 ^a^	105.01 ± 0.94 ^a^	104.59 ± 0.05 ^a^
Mn	0.86 ± 0.50 ^b^	0.88 ± 0.03 ^b^	0.95 ± 0.01 ^a^	0.97 ± 0.02 ^a^
Na	18.57 ± 0.66 ^c^	31.70 ± 0.87 ^b^	31.50 ± 0.79 ^b^	34.73 ± 0.67 ^a^
P	936.08 ± 2.69 ^a^	827.87 ± 4.94 ^b^	818.70 ± 2.68 ^c^	922.99 ± 9.77 ^a^
Zn	9.66 ± 0.26 ^a^	6.05 ± 0.20 ^d^	6.96 ± 0.07 ^c^	8.25 ± 0.27 ^b^

Values expressed in mg/100 g. Data are shown as the mean ± SD of three independent experimental runs. Different letters in each row represent significant differences at *p* < 0.05.

**Table 4:** Fatty acid composition (%) of partial substitution of avocado paste in corn chips.

Fatty Acid	Control (C)	C-2%	C-6%	C-10%
C8:0	0.040 ± 0.000 ^a^	0.022 ± 0.000 ^b^	0.029 ± 0.000 ^b^	0.023 ± 0.000 ^b^
C10:0	0.027 ± 0.000 ^a^	0.012 ± 0.000 ^c^	0.018 ± 0.000 ^b^	0.015 ± 0.000 ^c^
C12:0	0.051 ± 0.018 ^a^	0.023 ± 0.000 ^a^	0.027 ± 0.000 ^a^	0.029 ± 0.010 ^a^
C14:0	0.135 ± 0.007 ^a^	0.097 ± 0.000 ^b^	0.111 ± 0.00 ^b^	0.099 ± 0.000 ^b^
C14:1	ND	0.041 ± 0.010 ^a^	0.022 ± 0.000 ^a^	0.046 ± 0.010 ^a^
C15:0	ND	0.025 ± 0.000 ^a^	0.037 ± 0.020 ^a^	0.024 ± 0.010 ^a^
C16:0	14.228 ± 0.011 ^c^	14.624 ± 0.040 ^c^	15.274 ± 0.260 ^b^	16.143 ± 0.010 ^a^
C16:1	0.141 ± 0.018 ^d^	0.724 ± 0.000 ^c^	1.555 ± 0.100 ^b^	2.367 ± 0.030 ^a^
C17:0	0.083 ± 0.010 ^a^	0.073 ± 0.010 ^a^	0.087 ± 0.010 ^a^	0.076 ± 0.010 ^a^
C18:0	2.795 ± 0.0349 ^a^	2.452 ± 0.080 ^b^	2.266 ± 0.070 ^b,c^	2.126 ± 0.01 ^c^
C18:1	29.745 ± 0.109 ^d^	30.873 ± 0.360 ^c^	33.330 ± 0.260 ^b^	34.972 ± 0.000 ^a^
C18:2 (n-6)	50.551 ± 0.131 ^a^	48.891 ± 0.360 ^b^	45.033 ± 0.08 ^c^	41.892 ± 0.700 ^d^
C18:3 (n-3)	1.034 ± 0.017 ^d^	1.130 ± 0.000 ^c^	1.221 ± 0.010 ^b^	1.305 ± 0.000 ^a^
C18:3 (n-6)	0.516 ± 0.000 ^a^	0.487 ± 0.010 ^b^	0.444 ± 0.000 ^c^	0.401 ± 0.000 ^d^
C20:0	0.249 ± 0.037 ^a^	0.215 ± 0.010 ^a^	0.207 ± 0.010 ^a^	0.168 ± 0.030 ^a^
C20:5 (n-3)	0.155 ± 0.000 ^a^	0.096 ± 0.040 ^a^	0.146 ± 0.000 ^a^	0.128 ± 0.010 ^a^
C24:00	0.241 ± 0.000 ^a^	0.215 ± 0.000 ^b^	0.194 ± 0.000 ^c^	0.187 ± 0.000 ^c^
Omega-3 fatty acids	1.189 ± 0.017 ^a^	1.226 ± 0.046 ^a^	1.367 ± 0.005 ^a^	1.433 ± 0.011 ^a^
Omega-6 fatty acids	51.067 ± 0.131 ^a^	49.378 ± 0.362 ^b^	45.476 ± 0.088 ^c^	42.293 ± 0.070 ^d^
Omega-6/Omega-3 ratio	42.951 ± 0.518 ^a^	40.291 ± 1.208 ^a^	33.266 ± 0.184 ^b^	29.512 ± 0.172 ^c^
Total SFA	17.850 ± 0.032 ^b,c^	17.758 ± 0.038 ^c^	18.249 ± 0.224 ^b^	18.889 ± 0.040 ^a^
Total UFA	82.142 ± 0.021 ^a,b^	82.242 ± 0.038 ^a^	81.751± 0.224 ^b^	81.111 ± 0.040 ^c^
Total MUFA	29.887 ± 0.127 ^d^	31.638 ± 0.371 ^c^	34.908 ± 0.141 ^b^	37.385 ± 0.041 ^a^
Total PUFA	52.256 ± 0.149 ^a^	50.605 ± 0.408 ^b^	46.843 ± 0.083 ^c^	43.726 ± 0.081 ^d^
Atherogenic index (AI)	0.180 ± 0.000 ^c^	0.182 ± 0.000 ^c^	0.192± 0.003 ^b^	0.204 ± 0.000 ^a^
Thrombogenic index (TI)	0.389 ± 0.000 ^b^	0.388 ± 0.002 ^b^	0.398 ± 0.005 ^b^	0.415 ± 0.000 ^a^

SFA: saturated fatty acids; UFA: unsaturated fatty acids; MUFA: monounsaturated fatty acids; PUFA: polyunsaturated fatty acids; ND: not detected. Values expressed as g fatty acid/100 g oil. Data are shown as the mean ± SD of three independent experimental runs. Different letters in each row represent significant differences at *p* < 0.05.

**Table 5:** Phenolic compounds, total flavonoids, and antioxidant capacity of AP-supplemented corn chips.

Variable	Control (C)	C-2%	C-6%	C-10%
Total phenolic compounds	0.93 ± 0.09 ^a^	2.49 ± 0.22 ^b^	3.11 ± 0.23 ^c^	3.56 ± 0.35 ^d^
Total flavonoids	1.17 ± 0.22 ^a^	3.22 ± 0.29 ^b^	3.85 ± 0.24 ^c^	6.61 ± 0.41 ^d^
TEAC	2.28 ± 0.08 ^a^	4.22 ± 0.18 ^b^	4.31 ± 0.02 ^b^	5.27 ± 0.37
FRAP	1.19 ± 0.03 ^a^	1.90 ± 0.11 ^b^	2.07 ± 0.07 ^bc^	2.21 ± 0.24
DPPH	1.09 ± 0.02 ^a^	1.72 ± 0.17 ^b^	1.90 ± 0.17 ^c^	2.44 ± 0.19 ^d^
ORAC	7.81 ± 0.56 ^a^	20.08 ± 1.06 ^b^	21.52 ± 0.78 ^b^	22.52 ± 2.68 ^b^

Content of total phenolic compounds is expressed as mg GAE/g dw; total flavonoid content is expressed as mg QE/g dw; antioxidant activities are expressed as mg TE/g dw. Data are expressed as the mean ± SE of three independent experiments. Different letters in each row indicate significant differences at *p* < 0.05.

**Table 6:** Correlation matrix between phenolic compounds, flavonoids, and antioxidant capacities.

Variable	Phenolic Compounds	Flavonoids	ABTS	DPPH	ORAC
Flavonoids	0.945				
ABTS	0.973	0.942			
DPPH	0.878	0.948	0.910		
ORAC	0.997	0.891	0.976	0.835	
FRAP	0.990	0.947	0.973	0.881	0.980

All correlations listed are significant at *p* < 0.05.

## Data Availability

The original contributions presented in the study are included in the article, further inquiries can be directed to the corresponding author.
